# Expression, purification and crystallization of wheat profilin (Tri a 12)

**DOI:** 10.1186/2045-7022-1-S1-P4

**Published:** 2011-08-12

**Authors:** Margit Cichna-Markl, Peter Forstenlechner, Karin Hoffmann-Sommergruber, Dalibor Milić, Dubravka Matković-Čalogović, Christina Ecker

**Affiliations:** 1 University of Vienna, Department of Analytical Chemistry, Vienna, Austria; 2 Medical University of Vienna, Department of Pathophysiology and Allergy Research, Vienna, Austria; 3 University of Zagreb, Laboratory of General and Inorganic Chemistry, Zagreb, Croatia

## 

Wheat profilin, designated Tri a 12, has recently been found to be recognized by specific IgE antibodies in patients suffering from bakers' asthma, wheat induced food allergy and also in patients with grass pollen allergy. Since profilin sequences are highly conserved among plants, individuals who are sensitized to profilin commonly show allergic symptoms to a large number of unrelated plants. Knowledge about the three dimensional structure of these allergenic proteins is necessary to gain information on the surface structure required to predict cross-reactivity. However, in contrast to other plant profilins, the three dimensional structure of Tri a 12 has not been resolved yet.

In the present study Tri a 12 was expressed in E. coli and purified from the soluble fraction by affinity chromatography. The molecular weight and the structural integrity of the recombinant protein were verified by mass spectrometry and circular dichroism, respectively. Recombinant Tri a 12 showed intact secondary structures of mixed alpha helices and beta sheet elements characteristic for members of the profilin protein family. This well characterized batch of purified recombinant wheat profilin was then used for crystallization.

Crystallization conditions were screened with the sitting-drop vapour-diffusion method. The best crystals of wheat profilin with maximal dimensions between 0.1 and 0.3 mm were observed after one week at 290 K for reservoir solutions containing 3.2-3.7 mol L-1 sodium formate and 50 mmol L-1 HEPES–NaOH buffer, pH = 7.5. A data set diffracting to a resolution of 3.3 Å was collected in-house from a single crystal. The crystals belonged to space group P3221, with unit-cell parameters a = b = 58.9 Å, c = 82.5 Å, α = β = 90° and γ = 120°. Model building and refinement of the crystal structure, as well as further optimization of crystal diffraction quality is under way.

